# Assessing the Implementation and Awareness of Children’s Rights in Pediatric Hospital: A Comparative Study of Parents’ and Children’s Perspectives

**DOI:** 10.3390/pediatric17030064

**Published:** 2025-06-08

**Authors:** Vasiliki Georgousopoulou, Chrysoula Dafogianni, Pinelopi Vlotinou, Aspasia Serdari, Ioannis Koutelekos, Anna Tsiakiri, Dimitrios Cassimos, Maria Lavdaniti, Maria Amanatidou, Georgios Manomenidis

**Affiliations:** 1 Department of Nursing, Democritus University of Thrace, 68100 Alexandroupolis, Greece; vageorgo@nurs.duth.gr (V.G.); amanatidoumara@gmail.com (M.A.); gmanomen@nurs.duth.gr (G.M.); 2 Department of Nursing, Faculty of Health and Care Sciences, University of West Attica, 12243 Egaleo, Greece; cdafog@uniwa.gr; 3 Department of Occupational Therapy, University of West Attica, 12243 Egaleo, Greece; 4 Department of Child and Adolescent Psychiatry, Democritus University of Thrace, 68100 Alexandroupolis, Greece; aserntar@med.duth.gr; 5 Department of Nursing, University of West Attica, 12243 Egaleo, Greece; ikoutel@uniwa.gr; 6 Department of Neurology, Democritus University of Thrace, 68100 Alexandroupolis, Greece; atsiakir@med.duth.gr; 7 Department of Pediatric, Democritus University of Thrace, 68100 Alexandroupolis, Greece; dkasimos@med.duth.gr; 8 Department of Nursing, International Hellenic University, 57001 Thessaloniki, Greece; maria_lavdaniti@yahoo.gr

**Keywords:** children’s rights, parents, perspectives, comparative study, awareness

## Abstract

To evaluate and compare the awareness and implementation of children’s rights in pediatric hospital settings from the perspectives of parents and children, this study emphasizes ethical considerations in healthcare, focusing on communication practices, privacy, and participation rights. **Methods:** A cross-sectional study was conducted in the largest pediatric hospital in Greece between February and April 2023. A total of 250 parents and 150 children participated. Data were collected using a structured questionnaire assessing six domains of children’s rights: access to information, participation, privacy, non-discrimination, play and recreation, and parental support. Statistical analysis included chi-square tests for categorical variables, with significance set at *p* < 0.05. **Results**: Significant discrepancies were identified between parents and children in their awareness of children’s rights. Only 2.9% of children were aware of printed lists of rights, and 46.3% of parents reported not knowing of their existence (*p* = 0.005). Children evaluated communication practices, such as the use of child-friendly language, more positively than parents (*p* = 0.02). Parents reported higher satisfaction with explanations of health conditions, treatments, and medication side effects (*p* < 0.001). Regarding privacy, children rated room allocation and privacy practices during examinations less positively than parents (*p* = 0.02). **Conclusions:** The study highlights critical gaps in the communication and implementation of children’s rights within pediatric hospitals. Tailored interventions, including staff training, development of child-friendly materials, and policy adjustments, are necessary to bridge these gaps and ensure an ethically sound healthcare environment that prioritizes the rights and well-being of both children and their families.

## 1. Introduction

Ensuring optimal living conditions and protecting the rights of children are crucial elements for promoting their well-being, growth, and development across various settings, including family, school, and healthcare [1]. Over the last century, the understanding of childhood as a distinct life phase has evolved, leading to greater awareness of children’s unique needs and rights [2]. The first significant international recognition of these rights was the Geneva Declaration of the Rights of the Child (UN, 1924), which laid the foundational principles that have expanded since then into frameworks like the United Nations Convention on the Rights of the Child (UNCRC) ratified in 1989 and adopted by 193 nations, including Greece. The UNCRC has promoted essential rights for children, such as access to healthcare, freedom from discrimination, and the right to participate in decisions involving them [3]. Specific rights addressing hospitalized children were later detailed in the European Association for Children in Hospital Charter [4], which emphasized child-centered care, respect for privacy, and parental presence as integral components of pediatric healthcare [5]. Recently, there has been increasing scrutiny on whether pandemic-related restrictions infringed upon these rights, particularly regarding parental presence during hospitalization [6]. Evidence suggests that while precautions are necessary, blanket restrictions on visitation can conflict with a child’s right to family support, impacting their recovery and emotional stability [7]. Studies in multiple countries, including the United States and Italy, highlighted significant variability in hospital policies during COVID-19 and called for more consistent protection of children’s rights [8]. In Greece, the UNCRC’s principles were embedded in national law in 1992, through the ratification of the Convention on the Rights of the Child (Standard: Law 2101/1992), and specific provisions were made to uphold these rights in healthcare settings [9]. However, research indicates gaps in awareness and application, particularly among healthcare providers and parents in pediatric hospitals [10]. A study by Georgousopoulou et al. [11] assessed the respect for children’s rights within Greek hospitals, revealing challenges in fully implementing UNCRC standards, such as adequate communication, privacy, and parental presence during hospitalizations [1]. Findings from various countries, including Romania, Iran, and Italy, similarly highlighted insufficient knowledge and observance of children’s rights among healthcare professionals, underscoring the need for targeted training and policy enforcement [12,13]. The aim of this study was to highlight the depth of parents’ and children’s perspectives on children’s rights within a pediatric hospital setting, as well as to assess how closely these rights are upheld in accordance with the UNCRC during hospitalization.

## 2. Materials and Methods

### 2.1. Participants and Methods

A convenience sample of 250 parents and 150 children, recruited from Aghia Sofia Children’s Hospital, the largest pediatric hospital in Greece, participated in this cross-sectional study from February 2023 to April 2023. Paper posters were placed outside all the hospital departments, calling for participation in the study. Interested participants contacted the first author and received further explanation about the study’s aim. The completion time was approximately 15–20 min. A ballot box was placed in each department to collect the questionnaires (Appendix A).

### 2.2. Demographic Characteristics

The demographic characteristics questionnaire was constructed for the purpose of the study and included questions about gender, age, level of education, and marital status.

### 2.3. The Knowledge of Implementation and Awareness of Children’s Rights Scale

A 20-item scale was developed for the purpose of the study using questions from the “Self-evaluation Model and Tool on the Respect of Children’s Rights in Hospitals Scale”, ref. [14] and the European Association for Children in Hospital Charter questionnaire [15]. This new scale assesses participants’ knowledge of children’s rights and the respect for these rights during hospitalization across six domains: (a) the Right to Access Information—Assessing awareness and provision of information to children and families, (b) the Right to Participation—Measuring respect for children’s involvement in decision-making processes about their care, (c) the Right to Privacy—Evaluating practices to ensure the privacy of children during medical procedures and hospitalization, (d) the Non-Discrimination—Examining equitable treatment of children irrespective of age, gender, or background, (e) the Right in Play and Recreation—Investigating access to recreational activities and spaces that promote emotional well-being, and (f) the Parental and Family Support—Assessing the accommodation of family-centered care practices, such as parental presence. The Cronbach’s alpha of internal consistency for the study was 0.86, indicating good reliability of the scale in measuring these constructs.

For the purposes of statistical presentation, 10 out of the 20 questions were selected based on their relevance to the 6 predefined domains and the presence of statistically significant differences between parents and children. This approach was adopted to enhance clarity and avoid excessive length in Section 3 while still providing a representative view of all domains.

The methodology followed the structure of the original study by Migone et al. [15], which excluded children under the age of 9 and treated children aged 9–17 as a single analytical group. For comparability, our study adopted the same age framework.

Inclusion criteria required a minimum of 48 h of hospital stay before participation. Children with chronic conditions were excluded. While specific diagnoses were not systematically collected, most children were admitted for acute conditions such as respiratory or gastrointestinal infections or minor surgical interventions.

### 2.4. Ethics Approval of Research

The study was approved by the Ethics Committee of the Faculty of Nursing of the National and Kapodistrian University of Athens (reference number: 660/14-01-2020). Participants were informed of the study’s purpose and goals, and that they could withdraw from the study at any time. Those who agreed were asked to sign a consent form. Description of how confidentiality and anonymity would be protected was provided by a cover letter accompanied by the questionnaire. The study was conducted in accordance with the Declaration of Helsinki.

### 2.5. Statistical Analysis

Categorical variables are presented as frequencies and percentages, while continuous variables are described using the mean and standard deviation. The chi-square test was employed to compare categorical variables, with *p*-values less than 0.05 considered statistically significant. Data analysis was conducted using IBM SPSS Statistics version 21.0 (IBM Corp., Released 2012, Armonk, NY, USA).

## 3. Results

The study sample comprised a total of 400 participants: 250 parents and 150 children. The majority of the respondents were female, accounting for 74.5% of parents and 51.4% of children. The mean age varied by group, with children having a mean age of 10.5 (±4.5) years and parents 42.2 (±6.5) years, respectively. Most parents were married (82.6%) and held a tertiary education degree (56.6%). Demographic characteristics of parents and children are displayed in Table 1.

The scale evaluates six key domains related to children’s rights during hospitalization, as outlined in Table 2.

With respect to the existence of a list that includes children’s rights in hospital corridors and rooms, only 2.9% of the children were aware of its existence and almost half of the parents (46.3%) (*p* = 0.005). Similarly, regarding the use of easy-to-understand language from health professionals, a significant difference was noted in children who reported higher rates of positive responses (*p* = 0.02). Children also reported more positive experiences compared to parents when they were asked if they were encouraged to ask questions about their health (*p* = 0.01). However, parents displayed higher rates of positive experiences compared to children regarding explanations about children’s health conditions, treatments, or medication side effects (*p* < 0.001). Regarding the time allocated to develop a relationship between children/parents and healthcare professionals, children provided more positive evaluations compared to parents (*p*-value = 0.05). Regarding respect for gender in rooms with more than two beds, children reported higher rates of positive responses compared to parents (*p*-value = 0.0021). Finally, in the question about whether curtains are used to ensure privacy during examinations, parents evaluated this aspect more positively than children (*p*-value = 0.02) (Table 3).

## 4. Discussion

### 4.1. Right to Access Information

The findings of the present study provide a comparative look at parents’ and children’s awareness regarding the implementation of children’s rights in a pediatric hospital. In particular, regarding the availability of a printed list of children’s rights in hospital clinics, parents reported at a higher rate than the children that they “did not know” about the existence of such a list. This highlights potential differences in access to information and experiences between the two groups. Parents may lack sufficient access or awareness regarding these lists, possibly due to inadequate visibility of these materials in spaces they wait around. Conversely, children may have encountered this information through educational or supportive interventions within the hospital. This discrepancy in responses could also reflect the perception and significance that each group attributes to this information. This difference may also point to inadequate communication strategies aimed at informing parents. According to Coyne et al. [16], parents often lack sufficient awareness of children’s rights in hospital settings, primarily due to ineffective communication with hospital staff. In contrast, children who participate in supportive activities, such as educational interventions or group therapy sessions, appear to have greater opportunities to learn about their rights.

Another significant finding of the study is that children reported higher rates of positive responses regarding the use of understandable language by hospital staff. This suggests that children perceive the language used by healthcare professionals as more tailored to their needs compared to parents. Healthcare staff likely adopt simpler and more comprehensible language when addressing children, taking into account their age and cognitive level. This adaptation may contribute to children’s feeling that they better understand the information provided. In the study by Coyne et al. [17] it was highlighted that children experience better communication when healthcare professionals adjust their language to match the child’s age, employing simple phrases and avoiding medical jargon. On the other hand, parents may have higher expectations for communication with hospital staff, seeking more detailed explanations or clarifications, which could lead to lower satisfaction. Parents often focus more on the specifics of treatments or medical decisions, which require greater precision and elaboration from the staff. Additionally, parents may feel that insufficient time is allocated to explaining procedures, contributing to more negative perceptions of the communication style compared to children. This finding is in agreement with a previous study in which communication with parents was perceived as more complex since parents demand more extensive explanations to understand the nature of treatments and medical decisions. This complexity can amplify the perception that communication is not always adequate [18].

### 4.2. Right to Participation

The findings related to the encouragement of children to ask questions about their health condition, as well as the explanation of their health status by hospital staff, reveal notable patterns that highlight differences in perceptions and experiences between children and parents. Children reported more positive experiences regarding encouragement from hospital staff to ask questions about their health. This may be attributed to a child-centered approach adopted by healthcare professionals, who strive to create a supportive environment for children. According to Coyne et al. [16], active participation of children in discussions about their health enhances their autonomy and understanding of their condition. On the other hand, parents reported significantly higher rates of positive responses regarding the explanation of health conditions, treatments, or medication side effects by hospital staff. This is likely to reflect the priority given by healthcare professionals to provide detailed and comprehensive information to parents, who are often responsible for making care-related decisions. In addition, Coyne et al. [17] emphasized that parents frequently perceive detailed explanations of treatments as a critical factor for building trust and fostering collaboration with healthcare staff. Moreover, Lambert et al. [18] demonstrated that encouraging children to express questions or concerns is essential for improving their healthcare experience. Children who actively participate in discussions tend to feel greater trust in healthcare professionals and experience reduced anxiety. Similarly, Coyne et al. [17] underlined the importance of providing clear and detailed medical information to parents, highlighting that this empowers them and enables active participation in decision-making processes.

### 4.3. Parental and Family Support

Regarding the time allocated for building relationships between children/parents and healthcare professionals, children provided more positive evaluations of this aspect compared to parents. This finding suggests that children may perceive interactions with healthcare professionals differently than parents. Children often experience these interactions as more personal and supportive, especially when professionals adopt child-centered approaches, such as using understandable language and engaging in direct communication. The positive evaluation by children likely reflects a focus on the quality rather than the quantity of time spent. In contrast, parents tend to focus more on the adequacy of time devoted to explanations and decision-making processes. They may feel pressure to understand complex medical information or make critical decisions regarding their child’s care. This pressure may lead to a perception that the time allocated for communication is insufficient. According to Lambert et al. [18], parents frequently report the feeling that the time available for communication is limited. Insufficient time can exacerbate feelings of frustration or anxiety, particularly when parents seek detailed and precise explanations about treatments and care plans. Healthcare professionals may prioritize relationship-building with children to create a safe and supportive environment, which could explain why children report more positive experiences compared to parents. As noted by Lee et al. [19], adequate time for developing relationships between healthcare professionals and families is essential not only to address immediate medical needs but also to build trust and foster collaboration.

### 4.4. Non-Discrimination

Regarding the allocation of children to rooms based on age and gender, children provided more positive responses, reflecting potentially different perceptions or experiences between the two groups. Children likely evaluate room allocation primarily from a social perspective. Sharing a room with peers or children of the same gender may foster a sense of familiarity, safety, and companionship, positively influencing their perceptions. As noted by Coyne et al. [16], a sense of security in hospital rooms significantly impacts children’s overall experience. Grouping children with peers or those of the same gender has a positive effect on their psychology and helps reduce anxiety. In contrast, parents tend to focus more on factors such as privacy, safety, and the mental well-being of their child. If clear policies regarding room allocation based on age and gender are not apparent, parents may adopt a more critical perspective. This concern could stem from reports or experiences of inappropriate allocation. Coyne et al. [17] emphasized that parents often prioritize practical aspects of hospitalization, such as cleanliness, privacy, and allocation, whereas children focus more on their social relationships during their stay. It is also possible that room allocation policies are not adequately communicated to parents, leading to their less favorable perceptions. Meanwhile, children, focusing on their own experiences, tend to evaluate the environment more positively. According to Kokabisaghi et al. [20], adherence to the principles of the UNCRC requires room allocations that ensure the safety and well-being of children while also involving them actively in decisions about their environment.

### 4.5. Right to Privacy

Furthermore, the use of curtains to ensure privacy during medical examinations revealed that parents rated the use of curtains more positively than children. Parents appear to place greater emphasis on the importance of privacy, valuing curtains as a fundamental measure to protect the dignity and comfort of their child and family. This positive evaluation by parents may reflect their high expectations for upholding privacy standards in the hospital environment. According to Coyne et al. [16], privacy is a critical element in the hospital experience, especially for parents, who are often concerned about preserving their child’s dignity. The use of curtains or other protective measures is considered essential for fostering trust in the healthcare system. On the other hand, children may not perceive curtains as an adequate privacy measure. They might view curtains as insufficient barriers or place greater importance on the presence of other individuals in the room during examinations. Additionally, their age and level of understanding may influence their evaluation of the importance of privacy. Lambert et al. [18] noted that children may not value privacy to the same extent as adults unless they have experienced situations where they felt exposed. The significance they attribute to privacy often depends on their age and maturity level. Cultural and social perspectives on privacy may also play a role in shaping these differing perceptions. Parents, given their protective role, are likely more sensitive to privacy concerns. Meanwhile, children’s focus might be more situational and influenced by their immediate environment and interactions. Coyne et al. [17] highlighted the importance of ensuring privacy in busy hospital settings, recommending the use of physical barriers, such as curtains, along with practices that minimize the sense of exposure for patients. These measures can significantly enhance the perception of safety and comfort, particularly in shared spaces.

While the discussion addressed all six domains of the questionnaire, certain domains were more prominently represented than others. Specifically, the “Right to Access Information” and the “Right to Participation” were discussed in greater depth due to the presence of significant statistical differences between parents’ and children’s responses. On the other hand, domains such as the “Right in Play and Recreation” were underrepresented, primarily because the related questions yielded fewer distinctive or conclusive patterns. This uneven representation is not indicative of bias but rather reflects the empirical strength of the findings across domains. In future studies, efforts should be made to ensure a more balanced exploration of all domains.

## 5. Limitations

This study provides valuable insights into the perceptions and experiences of children and parents within the hospital environment. However, certain limitations must be acknowledged to provide a balanced interpretation of the findings. The sample size and the specific population studied may not be representative of broader demographic or cultural groups. This limitation restricts the generalizability of the results to other contexts or regions, as the experiences and perceptions captured in this study may differ in diverse healthcare settings. The reliance on self-reported data introduces the potential for response bias, as participants may have provided socially desirable answers rather than fully accurate reflections of their experiences. This reliance on subjective reporting limits the ability to validate findings against objective measures or observational data. The study’s cross-sectional design captures perceptions at a single point in time, which limits the understanding of how these perceptions might evolve over time or in response to changes in hospital policies and practices. A longitudinal approach would have been more effective in exploring temporal changes and long-term impacts. Additionally, the study was conducted in a specific hospital setting, which may not fully reflect the practices, policies, or resource availability of other healthcare institutions. Variations in organizational culture, staffing, and resources could influence the applicability of these findings to other contexts. External factors such as socioeconomic status, educational background, and previous healthcare experiences were not extensively explored, although they may play a significant role in shaping the perceptions and responses of participants. Finally, the study did not specifically address the potential impact of cultural or language barriers on communication and understanding, which could have provided additional depth to the findings. These limitations highlight the need for caution in interpreting the results and suggest avenues for future research to address these gaps and build on the study’s findings.

It is also important to note that this study focused exclusively on the rights of hospitalized children, as defined by the EACH Charter and the structure of the validated questionnaire. Broader domains—such as continuity of schooling or the right to suffer as little as possible—were not assessed, as they fall outside the immediate context of hospitalization and were not among the predefined aims of the research.

Although the children were treated as a single analytical group (aged 9–17), we acknowledge that developmental differences may exist within this age range. Future studies with larger samples could stratify children by age (e.g., <10 and >10 years) to explore potential differences in perceptions more accurately.

## 6. Conclusions

The lack of awareness among parents about children’s rights highlights the need for improved visibility and accessibility of related materials. Communication with parents requires clarity and sufficient detail to foster trust in medical decisions, supported by staff training and tailored communication tools. Children value the quality of interactions with healthcare professionals, while parents focus on the adequacy of time for explanations and decision-making. Balancing these needs requires optimizing time for meaningful and efficient interactions and early discussions during hospital admissions. Adopting a multifaceted approach that addresses the unique needs of both groups can enhance the overall hospital experience, fostering trust and collaboration. The findings of this study underscore the urgent need for strategic interventions aimed at improving the visibility and accessibility of information regarding children’s rights within hospital settings. Emphasis should be placed on training healthcare professionals to communicate effectively with both parents and children, ensuring clarity and empathy in all interactions. Additionally, the development of child-friendly educational materials that are visually engaging and age-appropriate is essential to enhance children’s understanding of their rights. Hospitals should also prioritize policies that promote privacy and equitable allocation of space, fostering a more inclusive and supportive environment for all patients. Further research is warranted to explore the long-term impacts of enhanced communication strategies and rights-focused practices on the overall hospital experience. These efforts can form the basis for robust health policies that integrate the principles of children’s rights into routine healthcare delivery, ensuring a more consistent and equitable approach across pediatric hospital settings.

## Figures and Tables

**Table 1:** Demographic characteristics of study participants.

Demographic Characteristics	Parents *n* (%)	Children *n* (%)
**Gender** Males (%)	64 (25.5)	73 (48.6)
Females (%)/	186 (74.5)	77 (51.4)
Age (years) *	42.2 ± 6.5	10.5 ± 4.5
**Marital status**		
Married	207 (82.6)	- -
Single/Divorced	44 (17.4)	
**Level of education**		
Secondary education	28 (11.3)	
Tertiary education	142 (56.6)	
Master’s/PhD	78 (31)	

* Values are expressed as mean ± standard deviation.

**Table 2:** Domains Evaluated in the Children’s Rights Scale.

Domain	Description
Right to Access Information	Assesses awareness and provision of information to children and families regarding their rights and medical care.
Right to Participation	Evaluates respect for children’s involvement in decision-making processes about their healthcare.
Right to Privacy	Ensures practices that protect children’s privacy during medical procedures and hospitalization.
Right to Non-Discrimination	Examines equitable treatment of children irrespective of age, gender, or background.
Right to Play and Recreation	Investigates access to recreational activities and spaces that promote emotional well-being.
Parental and Family Support	Assesses the accommodation of family-centered care practices, including parental presence and support.

**Table 3:** Statistical test” refers to a comparison of the frequency of responses to the relevant questions between the groups of children and parents.

Questions	Parents (*n* = 250)		Children (*n* = 150)		Statistical Test
	N	%	N	%	
Does the hospital have a printed list of the child’s rights in the clinics?					X^2^: 12.79 *p* = 0.005
Yes	5	1.9	4	2.9	
No	41	16.7	41	25.7	
In some clinics	14	5.6	-	-	
I don’t know	190	75.8	108	71.4	
Does the hospital staff use language or words that are understandable to you?					X^2^: 12.39 *p* = 0.02
Yes	208	83.3	141	94.3	
No	14	5.6	-	-	
I don’t know	28	11.1	9	5.7	
Does the hospital staff encourage the child to ask questions about his or her health condition?					X^2^: 8.3 *p* = 0.01
Yes	180	72.2	116	77.1	
No	46	18.5	13	8.6	
I don’t know	24	9.3	21	14.3	
Does the hospital staff explain to the child and parents the course of the health condition, or treatments or side effects of medications?					X^2^: 51.9 *p* = 0.001
Yes	227	90.7	93	61.9	
No	14	5.6	21	14.3	
I don’t know	9	3.7	36	23.8	
Is there enough time to develop a relationship between children/parents and health care workers?					X^2^: 31.4 *p* = 0.05
Yes	97	38.9	98	63.6	
No	51	20.4	20	13.5	
Some times	88	35.2	32	22.9	
I don’t know	14	5.5	-	-	
Does the hospital staff allocate children to rooms according to age and gender?					X^2^: 55.2 *p* = 6.1
Yes	42	16.7	39	25.7	
No	88	35.2	51	34.3	
Some times	46	18.5	47	31.4	
I don’t know	74	29.3	13	8.6	
Is there gender respect in double rooms or rooms with more than 2 beds?					X^2^: 70.9 *p* = 2.1
Yes	120	48.1	31	27.3	
No	46	18.5	91	60.6	
Some times	28	11.1	28	21.1	
I don’t know	56	22.2	-	-	
Are parents allowed to stay with their child at any time except when it is not in their child’s best interests?					X^2^: 5 *p* = 0.1
Yes	203	81.5	132	88.6	
No	19	7.4	9	5.7	
Some times	23	9.3	9	5.7	
I don’t know	5	1.9	-	-	
Does the hospital staff during the examination in rooms with more than one bed use curtains for privacy?					X^2^: 9 *p* = 0.02
Yes	153	61.1	85	57.1	
No	46	18.5	43	28.6	
Some times	32	13	9	5.7	
There was no other child in the room	19	7.4	13	8.6	
Are there adequate spaces for children’s play, education and entertainment?					X^2^: 82.2 *p* = 0.1
Yes	56	22.2	17	11.4	
No	92	37	64	42.9	
Some times	18	5.6	56	37.1	
I don’t know	84	32.2	13	8.6	

Note: “-”non-responses.

## Data Availability

No new data were created or analyzed in this study.

## Abbreviations

The following abbreviation is used in this manuscript:

UNCRC	United Nations Convention on the Rights of the Child

## Appendix A

**Knowledge of Implementation and Awareness of Children’s Rights Scale** The following questionnaire was developed to assess the knowledge and implementation of children’s rights in hospitals.
**Right to Access Information**
Does the hospital have a printed list of the child’s rights in the clinics?
☐ Yes
☐ No
☐ Sometimes
☐ I don’t know
Does the hospital staff explain to the child and parents the course of the health condition, treatments, or side effects of medications?
☐ Yes
☐ No
☐ I don’t know
Does the hospital staff use language or words that are understandable to you?
☐ Yes
☐ No
☐ I don’t know
**Right to Participation**
Does the hospital staff encourage the child to ask questions about his or her health condition?
☐ Yes
☐ No
☐ I don’t know
Are children given the opportunity to express their opinions about their treatment?
☐ Yes
☐ No
☐ Sometimes
☐ I don’t know
Are children included in decisions regarding their care?
☐ Yes
☐ No
☐ Sometimes
☐ I don’t know
**Right to Privacy**
Does the hospital staff use curtains for privacy during examinations in rooms with more than one bed?
☐ Yes
☐ No
☐ Sometimes
There was no other child in the room
Is information regarding the child’s health status kept confidential?
☐ Yes
☐ No
☐ Sometimes
☐ I don’t know
**Right to Non-Discrimination**
Does the hospital staff allocate children to rooms according to age and gender?
☐ Yes
☐ No
☐ Sometimes
Is there gender respect in double rooms or rooms with more than two beds?
☐ Yes
☐ No
☐ Sometimes
☐ I don’t know
Are children with disabilities provided with appropriate accommodations?
☐ Yes
☐ No
☐ Sometimes
☐ I don’t know
**Right to Play and Recreation**
Are there adequate spaces for children’s play, education, and entertainment?
☐ Yes
☐ No
☐ Sometimes
Are hospitalized children given the opportunity to participate in educational activities?
☐ Yes
☐ No
☐ Sometimes
☐ I don’t know
Does the hospital provide emotional support programs for children during hospitalization?
☐ Yes
☐ No
☐ Sometimes
☐ I don’t know
**Parental and Family Support**
Is there enough time to develop a relationship between children/parents and healthcare workers?
☐ Yes
☐ No
☐ Sometimes
☐ I don’t know
Are parents allowed to stay with their child at any time except when it is not in their child’s best interests?
☐ Yes
☐ No
☐ Sometimes
☐ I don’t know
Does the hospital provide facilities for parents to stay overnight with their child?
☐ Yes
☐ No
☐ Sometimes
☐ I don’t know
**Right to Protection from Harm**
Is the hospital environment safe and adapted to children’s needs?
☐ Yes
☐ No
☐ Sometimes
Are children protected from unnecessary medical procedures or treatments?
☐ Yes
☐ No
☐ Sometimes
☐ I don’t know
Does the hospital staff receive training on children’s rights and child-friendly healthcare practices?
☐ Yes
☐ No
☐ Sometimes
☐ I don’t know
